# Dementia subtype specification and cognitive evidence visibility in Turkish Court of Cassation decisions: a document analysis of 1,031 decisions (2005–2025)

**DOI:** 10.1186/s12910-026-01480-w

**Published:** 2026-05-21

**Authors:** Abdullah Güzel, Furkan İncebacak, Elif Simin Issı

**Affiliations:** Department of Neurology, Faculty of Medicine, Afyonkarahisar Health Sciences University, Afyonkarahisar, Türkiye

**Keywords:** Dementia, Dementia subtype specification, Cognitive evidence, Cognitive assessment, Legal capacity, Decision-making capacity, Medico-legal ethics, Judicial reasoning, Transparency, Court of Cassation, Document analysis

## Abstract

**Background:**

Dementia is frequently invoked in medico-legal contexts where questions of legal capacity, guardianship, testamentary validity, transactional validity, and criminal responsibility arise. Although subtype differentiation and cognitive assessment are central to clinical dementia evaluation, it remains unclear to what extent such clinically meaningful detail is visible in appellate judicial reasoning. This study examined the visibility of dementia subtype specification and cognitive evidence in decisions of the Turkish Court of Cassation (2005–2025), and examined the ethical implications of these textual patterns for transparency, accountability, and procedural fairness in judicial decision-making.

**Methods:**

We conducted a retrospective, cross-sectional, document-based analysis of publicly accessible Court of Cassation decisions. Using keyword-based searches (“demans,” “bunama,” and “Alzheimer”), 1,180 decisions were identified; after screening for legal relevance, 1,031 decisions were included. Each decision was coded across 15 variables, including dementia subtype specification, cognitive evidence visibility, legal context, and medical report source. Descriptive statistics were calculated for all variables. Temporal trends in dementia subtype visibility were analysed using logistic regression, supplemented by year-by-year distributional assessment and multivariable sensitivity modelling. A parallel logistic regression was also conducted for the broader binary outcome of any visible cognitive evidence (specific or non-specific; *n* = 104 events).

**Results:**

Dementia subtype information was explicitly specified in 46.4% of decisions (478/1,031; 95% CI, 43.3–49.5%), whereas 53.6% used only general or unspecified terminology. Alzheimer’s disease was the most frequently reported subtype, appearing in 451 decisions (43.7% of the full sample; 94.4% of subtype-specified decisions). Subtype visibility increased significantly over time (OR = 1.062 per year; 95% CI, 1.034–1.091; *p* < 0.001). In contrast, visible cognitive evidence remained limited: 89.9% of decisions contained no reference to cognitive assessment, 9.8% included only non-specific references, and only 0.3% explicitly named the Mini-Mental State Examination (MMSE; 95% CI, 0.1–0.8%). No decision referred to the Montreal Cognitive Assessment (MoCA). Any visible cognitive evidence was present in 10.1% of decisions (104/1,031; 95% CI, 8.3–12.1%) and was highest in guardianship/restriction cases (19.7%). Temporal modelling of any visible cognitive evidence was associated with a statistically significant decrease over the study period (OR = 0.918 per year; 95% CI, 0.880–0.958; *p* < 0.001).

**Conclusions:**

Dementia subtype specification became moderately more visible in appellate judicial reasoning over time, whereas the textual visibility of cognitive evidence remained limited. These findings concern the traceability of clinically relevant information within decision summaries rather than the adequacy of the underlying evidentiary process itself. Strengthening the structured reporting of diagnostic basis, relevant cognitive findings, and functionally relevant capacity-related findings in medico-legal documentation may support improved transparency and accountability in the translation of clinical evidence into legal reasoning.

**Supplementary Information:**

The online version contains supplementary material available at 10.1186/s12910-026-01480-w.

## Introduction

Dementia is a heterogeneous clinical syndrome characterized by progressive deterioration in cognitive functions, affecting activities of daily living and personal independence. As an umbrella diagnostic term, “dementia” encompasses conditions that differ in aetiology, affected cognitive domains, clinical course, neuropsychiatric profile, and prognosis. Accordingly, subtype differentiation in clinical neurology practice (e.g., Alzheimer’s disease, vascular dementia, Parkinson’s disease/Lewy body dementia spectrum disorders, and frontotemporal dementia) is of fundamental importance [1–5]. Subtype differentiation is not merely a classificatory exercise; it informs the interpretation of disease course, accompanying neurological and behavioural symptoms, and the possible implications of cognitive impairment in legally relevant settings.

Clinical assessment in suspected dementia includes detailed history taking, neurological examination, and cognitive evaluation. Brief instruments such as the Mini-Mental State Examination (MMSE) and the Montreal Cognitive Assessment (MoCA) are widely used in clinical practice and remain important screening tools [6–10]. However, such instruments are not equivalent to comprehensive neuropsychological assessment, and they are not direct measures of legal capacity. Their visibility in judicial texts may therefore indicate the presence of some cognitive evidence, but not, by itself, the adequacy of the evidentiary basis for a legal determination. For this reason, the present study distinguishes between the visibility of cognitive evidence in judicial summaries and the sufficiency of the underlying clinical assessment.

The medico-legal relevance of dementia extends beyond the presence of a diagnosis. In contexts such as legal capacity, testamentary capacity, banking and financial transactions, guardianship or restriction decisions, and criminal responsibility, legally relevant decision-making depends not only on diagnosis but also on a multidimensional evaluation of understanding, appreciation, reasoning, and communication of choice [11–15]. Capacity is therefore context-specific and time-specific; a diagnosis of dementia does not, by itself, establish incapacity. Different legal contexts may also require different kinds of clinical information, making the translation of medical evidence into legal language especially important.

Decisions of the Turkish Court of Cassation (Yargıtay) guide lower courts and contribute to settled case law. These decisions do not reproduce the full evidentiary record; rather, they summarise the elements deemed necessary for appellate legal reasoning. The extent to which clinically meaningful detail is visible in such summaries provides an analytic approach to examining how medical evidence is translated into judicial text. In particular, examining whether dementia subtypes are specified and whether any cognitive evidence is visible—whether specific or non-specific—may clarify the traceability of clinically relevant information within appellate reasoning.

This study quantitatively evaluates two related dimensions in coded Court of Cassation decisions from 2005 to 2025: (i) dementia subtype visibility and (ii) cognitive evidence visibility within decision texts. The primary research questions were: (1) How often are dementia subtypes explicitly specified in Court of Cassation decisions? (2) To what extent do decisions include any visible cognitive evidence, including either named tests or non-specific references to cognitive assessment? (3) How does dementia subtype visibility change over time, and how is cognitive evidence distributed across the study period? By addressing these questions, the study aims to characterise how clinically relevant neurological information becomes visible—or remains invisible—in appellate legal texts.

## Methods

### Study design

This study was a retrospective, cross-sectional, document-based empirical legal analysis based on a secondary dataset constructed through systematic review of the textual content of Court of Cassation decisions [16, 17]. The analytic strategy was based on structured quantitative coding, supplemented by a directed qualitative component to support interpretation [18, 19]. Because the study focused on textual visibility rather than the full evidentiary record, the unit of analysis was the published decision text rather than the underlying case file.

### Data source and search strategy

The dataset was compiled using the Court of Cassation’s publicly accessible decision search system (Yargıtay Karar Arama). Decision texts were searched using the keywords “demans,“"bunama,” and “Alzheimer,” and results included decisions containing at least one of these terms. This search identified a total of 1,180 Court of Cassation decisions dated between 2005 and 2025. Because one of the three primary search terms (“Alzheimer”) was subtype-specific, the retrieval strategy may have increased the likelihood of capturing Alzheimer-related decisions relative to decisions using alternative subtype terminology. In addition, no subtype-specific search terms corresponding to Lewy body dementia, frontotemporal dementia, or vascular dementia were included beyond what could be captured through the general terms “demans” and “bunama.” This issue may have introduced subtype-related retrieval bias and is addressed as a methodological limitation. The most recent years (2024–2025) may also be relatively underrepresented because of publication lag in the decision database.

To address potential retrieval bias and assess the completeness of the search strategy, an additional exploratory sensitivity search was conducted using subtype-specific keywords, including “Lewy”, “frontotemporal”, “frontal”, and “vascular dementia/vasküler demans”. This supplementary search did not identify any additional legally relevant Court of Cassation decisions beyond those captured by the primary search strategy. Manual inspection of retrieved decision texts was also performed to identify alternative expressions referring to cognitive impairment or dementia-related conditions. No systematically distinct terminology pattern was identified. These findings suggest that, although the inclusion of “Alzheimer” as a subtype-specific search term may increase the relative visibility of Alzheimer-related cases, the overall composition of the dataset is not substantially driven by keyword selection alone. Nevertheless, the possibility of subtype-specific retrieval bias and incomplete capture of alternative terminology is acknowledged as a methodological limitation.

### Inclusion and exclusion process

The 1,180 decisions obtained in the initial search were reviewed in detail based on their decision texts. Files were excluded when the keywords appeared only indirectly, secondarily, or contextually, and when references to dementia, bunama, or Alzheimer’s disease were not legally determinative for the merits of the case. Following this screening process, 1,031 Court of Cassation decisions in which a dementia diagnosis or allegation was assessed to be directly related to legal consequences—such as legal capacity, validity of transactions, criminal responsibility, guardianship, wills, banking, or property transactions—constituted the final sample.

### Coding framework and variables

The unit of analysis was the individual Court of Cassation decision. The final dataset was structured such that each row represented one decision, yielding a total of 1,031 decisions. A total of 15 variables were coded for each decision. The primary outcome, dementia subtype visibility, was coded as “specified” when the decision text explicitly named a clinically recognised subtype and as “general/unspecified” when only generic expressions such as “dementia” or “bunama” were used. Expressions suggesting cognitive decline without allowing confident subtype assignment were treated as ambiguous and were coded within the general/unspecified category. The second primary outcome, cognitive evidence visibility, was coded as: (i) no mention, (ii) non-specific cognitive assessment, or (iii) specific named test. “Non-specific cognitive assessment” included references such as “clinical assessment,“"neuropsychiatric assessment,“"neuropsychological assessment,“"psychometric assessment,” or “cognitive evaluation by a specialist,” without naming a specific instrument. Additional coded variables included Supreme Court unit, legal context, legal capacity assessment, source of medical report, notarial act, and decision outcome. The STROBE checklist was completed and is provided as Additional file 2 [20]. A full codebook with operational definitions, coding rules, and illustrative examples for all 15 variables is provided as Additional file 1.

### Coding procedure and reproducibility

Coding was performed using a prespecified coding framework developed prior to the final analytic phase. Two investigators jointly reviewed each decision and applied the coding criteria through real-time consensus. The coding framework included explicit operational definitions, predefined coding rules, and representative examples for all variables, which guided the coding process and minimised interpretive variability. Ambiguous cases were discussed in detail and resolved through consensus, with involvement of a third author when necessary. This iterative consensus-based approach was applied consistently across all decisions to ensure internal coherence in the interpretation of complex medico-legal texts.

Because coding was conducted through joint consensus rather than independent parallel coding, formal inter-rater reliability coefficients (e.g., Cohen’s kappa) were not calculated. While this approach enhances internal consistency, it limits the ability to quantify reproducibility across independent coders and is acknowledged as a methodological limitation.

### Statistical analysis

Descriptive statistics were reported as counts and percentages. For the primary outcome proportions, 95% binomial confidence intervals were calculated. Dementia subtype visibility across Supreme Court units and cognitive evidence visibility across legal contexts were explored using chi-square tests, with cautious interpretation where sparse cells were present. Temporal change in dementia subtype visibility was examined first using a univariable logistic regression model with decision year treated as a continuous predictor and then using a multivariable sensitivity model additionally adjusting for Supreme Court unit and legal context. Model fit was evaluated using McFadden pseudo-R² and Hosmer–Lemeshow statistics. For cognitive evidence visibility, a parallel temporal model was fitted using the broader binary outcome of any visible cognitive evidence (specific or non-specific combined; *n* = 104 events), which provided sufficient events for stable inference. Explicit named-test references alone (*n* = 3) were too sparse for separate temporal modelling and are reported descriptively. Analyses were performed using Python 3.x (pandas, scipy; logistic regression: statsmodels; confidence intervals: Wilson score method for proportions < 5%).

### Ethical considerations

This study is a document-based investigation conducted using publicly available and anonymised Court of Cassation decisions. There was no direct intervention involving human participants and no use or processing of personal data or special-category personal data. In line with applicable national and international research ethics principles, ethics committee approval was not required.

## Results

A total of 1,031 Court of Cassation decisions dated between 2005 and 2025 were analysed. The median decision year was 2018, and the interquartile range (IQR) was 2014–2022. The annual distribution of included decisions was markedly left-skewed: the lower half of cases spanned thirteen years (2005–2018), whereas the upper half spanned only seven years (2019–2025). Annual case counts ranged from 1 decision in 2005 to 106 decisions in 2024, with a marked increase in volume from 2012 onward. The most recent years showed the highest case volumes (2024: *n* = 106; 2023: *n* = 86; 2025: *n* = 44), although 2024–2025 may remain partially underrepresented because of publication lag in the online decision database.

### General characteristics of the decisions

Of the decisions, 82.6% (*n* = 852) were issued by civil chambers, 14.9% (*n* = 154) by criminal chambers, and 2.4% (*n* = 25) by general assemblies/other units (Table 1). In terms of legal context, the most frequent categories were “Unspecified/Other” (30.2%, *n* = 311) and “Sham transaction/Title deed” (20.2%, *n* = 208). These were followed by “Criminal responsibility/criminal” (14.7%, *n* = 152), “Guardianship/Restriction” (12.8%, *n* = 132), “Bank/Finance” (11.2%, *n* = 115), “Testament” (6.3%, *n* = 65), and “Legal capacity” (4.7%, *n* = 48) (Table 1).

**Table 1:** Basic characteristics of the sample (*n* = 1,031)

Variable	*n*	%
Supreme Court unit		
Civil chambers	852	82.6
Criminal chambers	154	14.9
General assembly / other	25	2.4
Case context		
Unspecified / Other	311	30.2
Sham transaction / Title deed	208	20.2
Criminal responsibility / criminal	152	14.7
Guardianship / Restriction	132	12.8
Bank / Finance	115	11.2
Testament	65	6.3
Legal capacity	48	4.7
Legal capacity assessment		
Contested	520	50.4
Present	195	18.9
Absent	291	28.2
Not stated	25	2.4
Source of medical report		
Forensic Medicine Institute (ATK)	484	46.9
Clinical source	158	15.3
Not stated	389	37.7
Notarial act		
Yes	164	15.9
No	848	82.3
Not stated	19	1.8
Decision outcome		
Reversal	524	50.8
Approval	446	43.3
Partial	29	2.8
Other	32	3.1

The “Unspecified/Other” category represented a heterogeneous residual grouping of legally relevant decisions that did not fit the six predefined focal categories. Analysis of the raw decision content within this category revealed several recurring sub-patterns. The largest sub-group comprised general capacity or incapacity disputes (ehliyetsizlik, fiil ehliyeti, hukuki ehliyet) that could not be confidently assigned to a more specific legal context, primarily because dementia was invoked as a subsidiary or contextual element rather than the primary basis of the claim (*n* = 114; 36.7% of the Unspecified/Other group). The second largest sub-group contained decisions involving mixed or atypical legal claims in which dementia featured incidentally, including compensation and disability cases (*n* = 24; 7.7%), fraud and misrepresentation disputes (*n* = 16; 5.1%), family law proceedings (*n* = 8; 2.6%), exploitation of cognitive impairment claims (*n* = 6; 1.9%), social security and labour disputes (*n* = 5; 1.6%), and procedural power-of-attorney or guardianship issues (*n* = 5; 1.6%). A residual group of 24 decisions (7.7%) could not be reliably sub-classified from the decision text alone. Cognitive evidence visibility within the Unspecified/Other category was the lowest of all categories at 1.9% (6/311), consistent with the subsidiary role of dementia in these cases.

### Dementia terminology and dementia subtype visibility

**Fig. 1:**
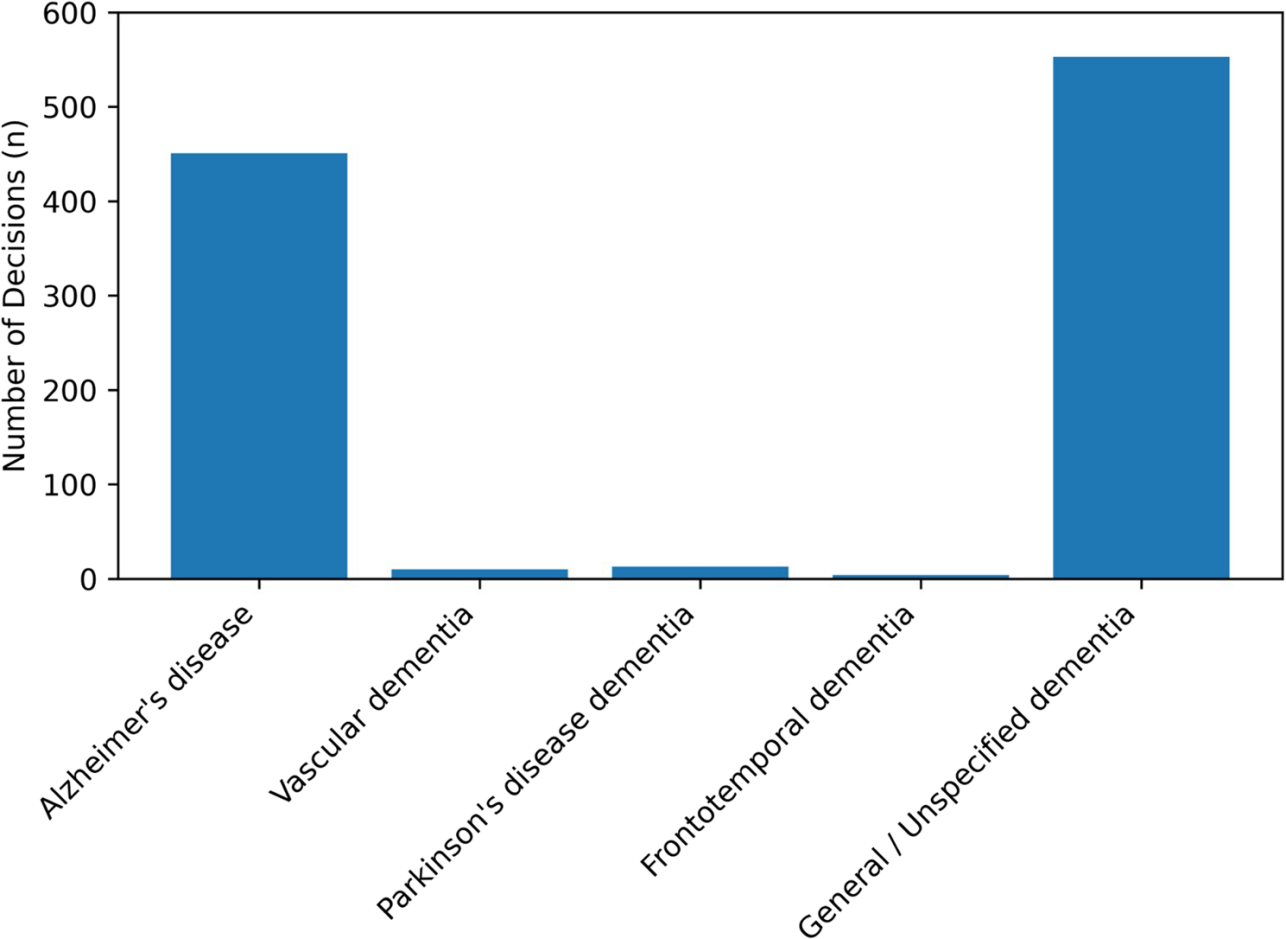
Distribution of dementia terminology and subtype visibility in 1,031 Turkish Court of Cassation decisions (2005–2025). The x-axis shows the dementia terminology category recorded in the decision text; the y-axis shows the number of decisions in each category (*n* = 1,031 total). Alzheimer’s disease was the most frequently specified subtype (*n* = 451; 43.7%), followed by Parkinson’s disease dementia (*n* = 13; 1.3%), vascular dementia (*n* = 10; 1.0%), and frontotemporal/frontal-type dementia (*n* = 4; 0.4%). General/unspecified dementia terminology was used in 553 decisions (53.6%)

When dementia-related terminology in the decisions was examined, Alzheimer’s disease was the most frequently reported subtype, appearing in 451 decisions (43.7% of the full sample; 94.4% of subtype-specified decisions). Vascular dementia appeared in 10 decisions (1.0%), Parkinson’s disease dementia/parkinsonism plus dementia in 13 decisions (1.3%), and frontotemporal/frontal-type dementia in 4 decisions (0.4%) (Table 2; Fig. 1).

**Table 2:** Dementia terminology and subtype distribution

Dementia term	*n*	%
Alzheimer’s disease	451	43.7
Vascular dementia	10	1.0
Parkinson’s disease dementia / parkinsonism plus dementia	13	1.3
Frontotemporal / frontal-type dementia	4	0.4
General dementia / unspecified (including ambiguous)	553	53.6
Total	**1**,**031**	**100.0**

“General dementia / unspecified” includes 378 clearly unspecified decisions, 100 ambiguous expressions, and 75 other non-standard or composite expressions that did not permit confident subtype assignment.

In contrast, 553 decisions (53.6%) did not explicitly specify a dementia subtype and used only general expressions such as “dementia/bunama” (Table 2). Accordingly, dementia subtype information was explicitly visible in 478 of 1,031 decisions (46.4%; 95% CI, 43.3–49.5%), whereas subtype information was not visible in 553 of 1,031 decisions (53.6%). Within the general/unspecified group, three coding sub-patterns were identified: clearly unspecified generic terminology (*n* = 378), ambiguous expressions suggesting but not permitting confident subtype assignment (*n* = 100), and other non-standard or composite expressions not mappable to a recognised subtype (*n* = 75). All three were retained under the operational category of general/unspecified because only explicit and unambiguous subtype naming was coded as specified.

By Supreme Court unit, dementia subtype visibility was 48.1% in civil chambers (410/852), 39.0% in criminal chambers (60/154), and 32.0% in general assemblies/other units (8/25) (Table 3). These differences were modest but statistically significant overall (χ² = 6.53, *p* = 0.038).

**Table 3:** Dementia subtype visibility by Supreme Court unit

Unit	Subtype specified, *n* (%)
Civil chambers	410/852 (48.1%)
Criminal chambers	60/154 (39.0%)
General assembly / other	8/25 (32.0%)

Overall comparison: χ² = 6.53, *p* = 0.038

When changes in dementia subtype visibility over time were evaluated, a significant increasing trend was observed. In the primary univariable logistic regression model, treating decision year as a continuous predictor, the odds of dementia subtype specification increased per year (OR = 1.062; 95% CI, 1.034–1.091; *p* < 0.001). Model fit was modest (McFadden pseudo-R² = 0.014), and the Hosmer–Lemeshow test suggested imperfect fit under a strictly linear assumption (χ² = 19.55, df = 8, *p* = 0.012). In a multivariable sensitivity model additionally adjusting for Supreme Court unit and legal context, the temporal association remained statistically significant and of comparable magnitude (adjusted OR = 1.055; 95% CI, 1.026–1.085; *p* < 0.001), with improved calibration (Hosmer–Lemeshow χ² = 8.49, df = 8, *p* = 0.387; McFadden pseudo-R² = 0.024). Given the uneven temporal distribution of cases, these findings indicate an overall descriptive increase rather than a smooth year-on-year pattern (see Additional file 3 for year-by-year distribution of decisions and outcomes).

### Visibility of cognitive evidence in decision texts

Visibility of cognitive evidence in decision texts was generally low. In 89.9% of decisions (n = 927), no visible reference to cognitive testing or cognitive assessment was present. In 101 decisions (9.8%), only a non-specific reference to cognitive assessment/testing (e.g., “clinical assessment,“"neuropsychiatric assessment,” “neuropsychological assessment,” or “psychometric assessment”) was included, without naming a specific test. A standardised cognitive test was explicitly named in only 3 decisions (0.3%; 95% CI, 0.1–0.8%), and these references were limited to the MMSE (Table 4). No visible reference to the MoCA was identified in the analysed decisions.

**Table 4:** Visibility of cognitive evidence in decision texts

Category	*n*	%
No mention	927	89.9
Non-specific cognitive assessment	101	9.8
Specific named test (MMSE)	3	0.3
MoCA	0	0.0

Accordingly, any visible cognitive evidence—specific or non-specific—was present in 10.1% of decisions (104/1,031; 95% CI, 8.3–12.1%). An exploratory review for standalone numerical cognitive scores identified only one decision containing a score pattern without broader standardised test reporting (an MMSE score of 23/30 in a procedural notification dispute), suggesting that numerical score visibility without full instrument contextualisation was also exceptionally rare. A logistic regression for the temporal trend in any visible cognitive evidence (binary: present vs. absent; *n* = 104 events) yielded OR = 0.918 per year (95% CI, 0.880–0.958; *p* < 0.001; McFadden pseudo-R² = 0.023), indicating a statistically significant decrease over the study period. Year-by-year inspection showed that non-specific cognitive assessment references were more frequent in the 2008–2015 period (10–31% of annual decisions) than in the 2016–2025 period (2–13%), corresponding to the observed declining trend (Additional file 3). Explicit named-test references alone (*n* = 3) were too sparse for separate temporal modelling.

**Fig. 2:**
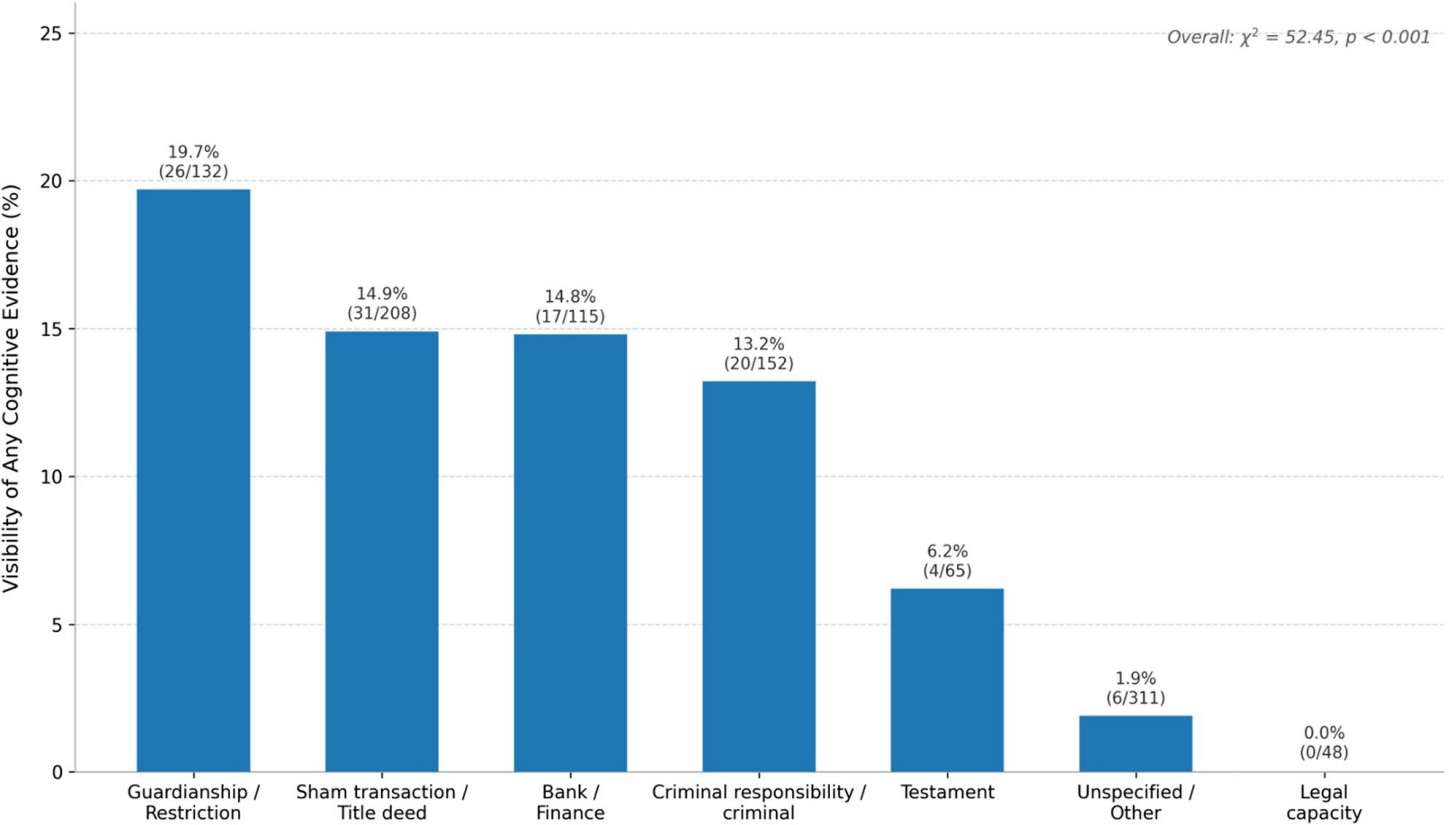
Visibility of any cognitive evidence by legal context in 1,031 Turkish Court of Cassation decisions (2005–2025). Values represent the percentage of decisions within each legal context containing either a specific named cognitive test or a non-specific reference to cognitive assessment. Overall comparison: χ² = 52.45, *p* < 0.001

Decisions containing any reference to cognitive assessment/testing varied by legal context. Cognitive evidence visibility was highest in guardianship/restriction files at 19.7% (26/132). This was followed by sham transaction/title deed cases at 14.9% (31/208), bank/finance cases at 14.8% (17/115), and criminal responsibility/criminal cases at 13.2% (20/152). The rate was 6.2% in testament cases (4/65) and 1.9% in the unspecified/other category (6/311). No cognitive assessment/test reference was identified in the legal capacity category (0/48; 0.0%) (Table 5; Fig. 2). Overall differences across legal contexts were statistically significant (χ² = 52.45, *p* < 0.001), although sparse cells in some categories warrant cautious interpretation.

**Table 5:** Visibility of cognitive evidence by case context

Case context	*n*/*N*	%
Guardianship / Restriction	26/132	19.7
Sham transaction / Title deed	31/208	14.9
Bank / Finance	17/115	14.8
Criminal responsibility / criminal	20/152	13.2
Testament	4/65	6.2
Unspecified / Other	6/311	1.9
Legal capacity	0/48	0.0

Overall comparison: χ² = 52.45, *p* < 0.001. Sparse cells in some categories warrant cautious interpretation

### Legal capacity, medical source, and decision outcome

Legal capacity assessment was coded as “contested” in 50.4% of decisions (*n* = 520). Decisions indicating that capacity was present accounted for 18.9% (*n* = 195), while those indicating that capacity was absent accounted for 28.2% (*n* = 291). Capacity status was not stated in 2.4% of decisions (*n* = 25) (Table 1).

With respect to the source of medical reports or diagnoses, references to the Forensic Medicine Institute (Adli Tıp Kurumu; ATK) appeared in 484 decisions (46.9%), whereas reports based on clinical sources (psychiatry, neurology, geriatrics, medical boards, etc.) appeared in 158 decisions (15.3%). In 37.7% of decisions (*n* = 389), the source of the medical report was not explicitly specified (Table 1). Regarding outcomes, reversals were the most frequent result at 50.8% (*n* = 524). Approvals accounted for 43.3% (*n* = 446), partial decisions for 2.8% (*n* = 29), and other outcomes for 3.1% (*n* = 32) (Table 1).

## Discussion

This study examined how clinically relevant dementia-related information becomes visible in appellate judicial summaries by analysing 1,031 Turkish Court of Cassation decisions issued between 2005 and 2025. Three main findings emerged. First, dementia subtype specification was visible in less than half of decisions, indicating that clinically meaningful diagnostic differentiation was not consistently reflected in appellate texts. Second, any visible cognitive evidence was uncommon, and explicit naming of standardised instruments was exceptionally rare. Third, dementia subtype visibility increased over time, whereas cognitive evidence remained limited across the corpus, indicating an uneven translation of clinical detail into legal language.

Because dementia is a heterogeneous syndrome, subtype differentiation matters clinically and may also matter medico-legally [1–5]. The distinction between Alzheimer’s disease, vascular dementia, Lewy body/Parkinson’s disease dementia spectrum disorders, and frontotemporal dementia is relevant not only to aetiology and prognosis but also to symptom profile, behavioural manifestations, and functional implications. In the present study, subtype specification was visible in 46.4% of Court of Cassation decisions. This pattern suggests that appellate summaries do sometimes preserve clinically meaningful diagnostic detail, but not in a consistent or institutionalised manner. Where subtype information is not visible in the decision text, the clinical specificity of the underlying evidence cannot be determined from the appellate summary alone. The present findings therefore support a description of incomplete textual traceability rather than a direct conclusion about the adequacy of the underlying clinical or judicial process.

Differences across Supreme Court units should also be interpreted cautiously. Subtype visibility was lower in criminal chambers than in civil chambers. This may be related to differences in drafting conventions, the structure of criminal reasoning, or the way medical evidence is summarised in different procedural settings. However, because the analysis was limited to Court of Cassation decision texts rather than full case files, these differences cannot be interpreted causally. At most, they point to possible structural variation in the mediation of clinical detail across legal domains.

The second major finding concerns cognitive evidence visibility. Brief screening instruments such as the MMSE and MoCA are widely used in clinical practice and may contribute to standardised communication about cognitive status [8–10]. At the same time, they are not equivalent to comprehensive neuropsychological assessment, and they are not direct proxies for legal capacity [21, 22]. For that reason, the current study does not interpret the absence of MMSE or MoCA naming as proof that the evidentiary basis of the case was inadequate. Rather, it shows that cognitive evidence was rarely visible in the appellate summary itself. The dataset captures textual visibility, not the full evidentiary record, and a lack of explicit instrument naming in the decision text may indicate omission from the summary, variability in reporting practices, or a more general absence of structured reporting in the medical documents reaching the court. Prior work has also suggested that the relationship between MMSE performance and decisional capacity is weak once shared verbal demands are accounted for [22]. In appellate decisions, a raw score without methodological context may therefore be interpreted as creating a misleading impression of evidentiary objectivity rather than genuine explanatory clarity. The present findings support a claim about limited traceability of cognitive evidence, not a claim that named screening tests would by themselves constitute sufficient evidentiary grounding.

This distinction becomes even more important when legal capacity is considered. Capacity is context-specific, multidimensional, and time-sensitive [11–15]. Guardianship, testamentary capacity, transactional validity, and criminal responsibility do not require identical clinical evidence, nor do they rely on the same functional thresholds. In this study, cognitive evidence visibility varied substantially across legal contexts and was unexpectedly absent in the legal-capacity category itself. This finding should not be read as proof that cognition was ignored in such cases; rather, it indicates that the manner in which clinically relevant information is summarised may differ substantially across legal settings. Future work should therefore attend more closely to the heterogeneity of legal capacity constructs rather than treating “capacity” as a single, uniform domain.

The ethical significance of these findings lies not in demonstrating a proven defect in judicial practice, but in identifying textual patterns that may matter for transparency, accountability, and procedural fairness. When individuals with dementia are involved in proceedings affecting autonomy, property, guardianship, or responsibility, the visibility of clinically meaningful reasoning may be relevant to how understandable and reviewable the legal justification appears. This concern is especially salient in light of rights-based frameworks emphasising equal recognition before the law, including Article 12 of the Convention on the Rights of Persons with Disabilities [23]. Recent international work has also highlighted divergences between medical and legal understandings of guardianship-related capacity in dementia [24]. The present study contributes to that conversation by focusing not on the substantive correctness of decisions, but on the textual traceability of the clinical information that appears within them.

Another underexplored finding concerns the source of medical reports. Nearly half of the decisions referred to the Forensic Medicine Institute (ATK), whereas more than one third did not clearly identify the source of the medical report at all. This pattern indicates that the visibility gap observed in appellate texts may be associated with both judicial summarisation practices and institutional reporting practices upstream of the court. If medico-legal reports vary in how explicitly they document subtype, cognitive findings, and functional implications, then appellate summaries may inherit that variability. The ATK therefore deserves greater attention in future work as a potential institutional mediator between clinical evidence and judicial text. From a practical perspective, the most immediate leverage points for reform may lie with forensic experts, the Forensic Medicine Institute, the Forensic Medicine Specialty Board, and judicial/expert-reporting standards that could support the development of clearer minimum reporting elements.

The temporal analysis also warrants restraint. A significant increase in subtype visibility was observed over time (OR = 1.062; 95% CI, 1.034–1.091; McFadden pseudo-R² = 0.014), which may be associated with growing diagnostic awareness, wider use of subtype-based classifications, or evolving reporting practices. However, because the distribution of cases across years is uneven, the early years are thinly populated, and the underlying change may be discontinuous rather than linear, this result should be interpreted as indicating an overall descriptive increase rather than proof of a smooth year-on-year institutional effect. The multivariable sensitivity model showed that the temporal association remained statistically significant after adjustment for court unit and legal context (adjusted OR = 1.055; 95% CI, 1.026–1.085; *p* < 0.001; Hosmer–Lemeshow *p* = 0.387; McFadden pseudo-R² = 0.024). A parallel logistic regression for any visible cognitive evidence (*n* = 104 events) yielded OR = 0.918 per year (95% CI, 0.880–0.958; *p* < 0.001; McFadden pseudo-R² = 0.023), indicating a statistically significant decrease. Year-by-year inspection indicates higher non-specific assessment references in the 2008–2015 period (10–31% of annual decisions) than in subsequent years (2–13%), rather than a uniform decline. Taken together, the two temporal trends move in opposite directions: dementia subtype visibility increased over time while any visible cognitive evidence decreased, indicating that increased diagnostic labelling in appellate texts was not accompanied by a corresponding increase in visible cognitive evidentiary documentation.

This study has several limitations. First, the analysis was based on published Court of Cassation decision summaries rather than full case files; absence from the text cannot be equated with absence from the evidentiary record. Second, the retrieval strategy relied on three Turkish search terms, including one subtype-specific term (“Alzheimer”), which may have introduced subtype-related retrieval bias. Although additional exploratory searches using subtype-specific terms (e.g., “Lewy”, “frontotemporal”, “vascular dementia”) did not yield additional eligible cases, the possibility of terminology-related retrieval bias cannot be entirely excluded. Third, the most recent years may be relatively underrepresented because of publication lag in court databases. Fourth, coding of free-text judicial material inevitably involves interpretive judgment, and no formal inter-rater reliability assessment was performed. Coding was performed jointly by two investigators through real-time consensus using predefined coding rules. Fifth, explicit named-test references were extremely rare, which limited stable inferential analysis for that narrow outcome. Sixth, although the annual distribution and sensitivity analyses strengthen temporal interpretation, the underlying change may still be non-linear and institutionally clustered. Finally, the study is situated in the Turkish legal system, and the transferability of its findings to other jurisdictions should be approached cautiously. The study does not allow inference about the adequacy or correctness of judicial decisions.

Taken together, the findings indicate that clinically relevant evidence is only partially visible in appellate dementia-related case law. The central issue is not simply whether a test name appears in a judgment, but whether legally meaningful clinical information can be traced in a way that allows the relationship between diagnosis, cognition, function, and legal reasoning to be understood.

## Conclusions

In this study, 1,031 Court of Cassation decisions from 2005 to 2025 were examined to assess the visibility of dementia subtype specification and cognitive evidence in appellate judicial summaries. Dementia subtype visibility was moderate and increased over time, whereas visible cognitive evidence remained limited and explicit naming of standardised instruments was rare.

These findings should be interpreted as relating to textual traceability rather than the full adequacy of the underlying evidentiary process. Nevertheless, the textual visibility of clinically meaningful information remains inconsistent. A more structured medico-legal reporting approach—one that clearly states the diagnostic basis, documents relevant cognitive findings, and relates functional findings to the specific legal construct under evaluation—may support improved traceability between clinical evidence and judicial reasoning.

Such improvements may require action not only by courts but also by the institutional actors who generate and transmit medico-legal evidence, including forensic experts, the Forensic Medicine Institute (ATK), the Forensic Medicine Specialty Board, and those responsible for judicial and expert-reporting standards.

## Supplementary Information

Additional file 1.

Additional file 2.

Additional file 3.

## Data Availability

All analyzed decisions are publicly accessible through the official Decision Search System of the Presidency of the Court of Cassation (Yargıtay Karar Arama). As the study relies solely on publicly available judicial documents, no additional datasets were generated beyond the coded analytical framework described in the Methods section. The coding framework may be made available by the corresponding author upon reasonable request.
