# Supplementary material for: Dementia subtype specification and cognitive evidence visibility in Turkish Court of Cassation decisions: a document analysis of 1,031 decisions (2005–2025)
Source: BMC Med Ethics. 2026 May 21;27:161. doi: 10.1186/s12910-026-01480-w (PMC13464883; doi:10.1186/s12910-026-01480-w)
Supplement: Supplementary file 1 [file 12910_2026_1480_MOESM1_ESM.docx]

**Supplementary File 1**

**CODEBOOK — Coding Manual for Variable Definitions, Categories, and Decision Rules**

*Dementia Subtype Specification and Cognitive Evidence Visibility in Turkish Court of Cassation Decisions: A Document Analysis of 1,031 Decisions (2005–2025)*

Abdullah Güzel, Furkan İncebacak, Elif Simin Issı

*Afyonkarahisar Health Sciences University, Faculty of Medicine, Department of Neurology, Afyonkarahisar, Türkiye*

**1. Overview and Purpose**

This codebook provides the operational definitions, coding categories, decision rules, and illustrative examples for all 15 variables coded in the dataset underlying this study. It serves two purposes: (a) to enable transparent evaluation of the coding procedure by peer reviewers and editors; and (b) to facilitate replication or extension of the study by future researchers.

The dataset comprises 1,031 Turkish Court of Cassation decisions issued between 2005 and 2025, identified through a keyword-based search of the official Yargıtay Karar Arama system. Each decision constitutes one unit of analysis.

**2. Dataset Structure**

| Unit of analysis | One Court of Cassation decision (n = 1,031) |
| --- | --- |
| Number of variables | 15 (V01–V15) |
| Primary outcome variables | V07 (Dementia Subtype Visibility) and V09 (Cognitive Evidence Visibility) |
| Data source | Yargıtay Karar Arama (https://karararama.yargitay.gov.tr/) |
| Study period | 2005–2025 (median year: 2018; IQR: 2014–2023) |

**3. Coding Procedure and Reproducibility**

Coding was conducted using a prespecified framework developed prior to the final analytic phase. Two investigators jointly reviewed each decision and applied coding rules through real-time consensus. The coding framework included explicit operational definitions, predefined coding rules, and representative examples for all variables.

Ambiguous cases were discussed in detail and resolved through consensus, with involvement of a third author when necessary. Disagreements and resolutions were documented in V15 (Notes).

**Limitation:** Because coding was performed through joint consensus rather than independent parallel coding, formal inter-rater reliability coefficients (e.g., Cohen’s kappa) were not calculated. This is acknowledged as a methodological limitation in the manuscript. Future studies should employ prospective dual-coder designs with reported kappa statistics.

**4. Search Strategy and Inclusion/Exclusion Criteria**

Three Turkish-language keywords were used: “demans” (dementia), “bunama” (a Turkish colloquial/clinical term for dementia/senility), and “Alzheimer.”

Decisions were included if the keyword was central to the legal determination — i.e., dementia was directly relevant to legal capacity assessment, transaction validity, criminal responsibility, guardianship, or a related medico-legal determination. Decisions were excluded if the keyword appeared only incidentally (e.g., in a witness list or unrelated procedural context). Exclusion decisions were documented in V15.

**Potential retrieval bias:** The inclusion of “Alzheimer” as a keyword (but not equivalent terms for other subtypes such as Lewy body dementia or frontotemporal dementia) may have introduced retrieval bias toward Alzheimer’s disease cases. An additional exploratory sensitivity search using subtype-specific keywords (“Lewy”, “frontotemporal”, “frontal”, “vascüler demans”) did not identify additional eligible cases. This limitation is acknowledged in the manuscript.

**5. Variable Definitions (V01–V15)**

Each variable is presented with: variable type, operational definition, categories/values (where applicable), coding rules, illustrative examples, and missing data handling.

| **V01 — Supreme Court Unit and Year** | |
| --- | --- |
| Variable type | Text (free entry) |
| Definition | Court unit (chamber name and number) and year of the decision, extracted directly from the decision header. |
| Coding rules | Court name and year taken from decision heading or first paragraph. Format: “Xth Civil Chamber YYYY” or “Xth Criminal Chamber YYYY.” Year coded as 4-digit YYYY. |
| Examples | ’17th Civil Chamber 2015’; ’3rd Civil Chamber 2021’; ’Criminal General Assembly 2019’ |
| Notes | Decision year used as continuous predictor in logistic regression (2005–2025). |
| Missing data | Cases with unidentifiable year excluded from trend analysis only. |

| **V02 — First Instance Court** | |
| --- | --- |
| Variable type | Text (free entry) |
| Definition | Type of lower court whose decision was subject to appellate review. |
| Coding rules | Court type recorded as stated in the decision text. Full name used if available; court type used if only partially identified. |
| Examples | Civil Court of First Instance; High Criminal Court; Civil Court of Peace |
| Notes | Descriptive variable; not used in primary analyses. |
| Missing data | Left blank if not stated. |

| **V03 — Plaintiff Role** | |
| --- | --- |
| Variable type | Text (anonymised) |
| Definition | Legal role of the initiating party. Personal identifying information anonymised; only role recorded. |
| Coding rules | Real names not coded. Party role entered: “Plaintiff” / “Heirs” (civil); “Claimant/Complainant” (criminal); “Guardian/Relative” (guardianship). |
| Examples | Plaintiff; Plaintiffs; Heirs; Claimant |
| Notes | No personal data recorded. Ethics: not applicable. |
| Missing data | N/A |

| **V04 — Defendant Role** | |
| --- | --- |
| Variable type | Text (anonymised) |
| Definition | Legal role of the opposing party. Same anonymisation rules as V03. |
| Coding rules | Same rules as V03. |
| Examples | Defendant; Defendants; Bank; Insurance company |
| Missing data | N/A |

| **V05 — Case Type** | |
| --- | --- |
| Variable type | Text (free entry) |
| Definition | Legal nature of the case — civil/criminal classification and primary claim type. |
| Coding rules | Primary claim coded from decision text. Multiple claims: primary claim coded; secondary claims noted in parentheses. |
| Examples | Title deed annulment and registration; annulment of will; guardianship; compensation |
| Notes | Forms the basis of legal context classification together with V06. |
| Missing data | Left blank if not determinable. |

| **V06 — Legal Context Category** | |
| --- | --- |
| Variable type | Categorical |
| Definition | Legal context of the case. Created to classify the medico-legal role of dementia in the decision. |

| **Category** | **Description** |
| --- | --- |
| **Guardianship / Restriction** | Cases involving placement under guardianship or legal restriction pursuant to Turkish Civil Code Art. 405 et seq. |
| **Testament** | Cases involving the validity, annulment, or enforcement of wills. |
| **Sham transaction / Title deed** | Cases involving fraudulent conveyance by a testator (muris muvazası), deed cancellation, and deceptive transfers. |
| **Bank / Finance** | Cases involving validity of banking transactions, negotiable instruments, mortgages, and guarantees. |
| **Criminal responsibility / criminal** | Cases involving defendant’s or victim’s criminal capacity (TCK Art. 32) or exploitation of cognitive impairment. |
| **Legal capacity** | Cases directly and primarily concerning a person’s legal capacity to act (capacity to perform legal transactions) as the central legal issue. |
| **Unspecified / Other** | Decisions not fitting the above categories: general legal incapacity disputes without a clearly assignable context, mixed claims, or unclassifiable cases. |
|  | |

| Coding rules | Primary legal context coded from decision. If two contexts equally present, both noted with a dash separator (e.g., “Muvazaa / Ehliyetsizlik”). Cases genuinely unclassifiable coded as Unspecified/Other with explanation in V15. |
| --- | --- |
| Missing data | Unclassifiable cases coded as Unspecified/Other. |

| **V07 — Dementia Subtype Visibility (Primary Outcome 1)** | |
| --- | --- |
| Variable type | Categorical (primary outcome variable 1) |
| Definition | Whether the decision text explicitly names a clinically recognised dementia subtype. This variable answers Research Question 1. |

| **Value** | **Coding criterion** |
| --- | --- |
| **Alzheimer’s disease** | Decision text explicitly states “Alzheimer,” “Alzheimer hastalığı,” or equivalent. |
| **Vascular dementia** | Decision text explicitly states “vascular dementia,” “cerebrovascular dementia,” or equivalent (including Turkish equivalents such as “vasküler demans”). |
| **Frontotemporal / frontal-type dementia** | Decision text explicitly states “frontal-type dementia,” “frontotemporal dementia,” or equivalent (including Turkish equivalents such as “frontal tip demans”). |
| **Parkinson’s disease dementia** | Decision text attributes dementia to Parkinson’s disease or uses equivalent terminology. |
| **General / unspecified** | Decision text uses only generic expressions (e.g., “dementia” or equivalent Turkish terms) without specifying a recognised subtype. Includes ambiguous and composite expressions. |
|  | |

| Coding rules | ONLY expressions actually used in the decision text are coded; coder’s own medical interpretation is never added. If “Alzheimer” and generic dementia terminology both appear, context determines whether Alzheimer is used as a subtype label or a generic reference. Ambiguous cases coded as General/unspecified with note in V15. Claim-level subtype statements not supported by a medical report noted as “(claimed)” in V15. |
| --- | --- |
| Study-wide statistic | 478/1,031 (46.4%; 95% CI 43.3–49.5%) coded as specified. |
| Missing data | Decisions without dementia reference excluded at screening; not applicable here. |

| **V08 — Legal Capacity Status** | |
| --- | --- |
| Variable type | Categorical (4 levels) |
| Definition | Whether the decision text records a finding on the legal capacity of the person with dementia at the time of the transaction or act under review. |
| Values | Present \| Absent \| Contested \| Not stated |
| Coding rules — Present | ATK or court explicitly determines capacity exists, or incapacity claim is rejected. |
| Coding rules — Absent | ATK report or court explicitly finds incapacity, or a prior final ruling establishes incapacity. |
| Coding rules — Contested | Court reversed the lower court decision due to insufficient examination, contradictory reports, or an unresolved capacity question. |
| Coding rules — Not stated | Capacity question not directly at issue in the decision (e.g., accident compensation cases) or not discernible from text. |
| Important | In criminal decisions, “sanık ehliyeti” (defendant) and “mağdur ehliyeti” (victim) are conceptually distinct; role noted in V15. |
| Coding reference point | Transaction or act date (not a future restriction decision). |
| Missing data | Same as V07. |

| **V09 — Cognitive Evidence Visibility (Primary Outcome 2)** | |
| --- | --- |
| Variable type | Categorical (primary outcome variable 2 — 3 levels) |
| Definition | Presence of any visible cognitive evidence in the decision text, whether a specifically named standardised instrument or a non-specific reference to cognitive assessment. This variable answers Research Question 2. |

| **Value** | **Coding criterion** | **Illustrative text** |
| --- | --- | --- |
| **Specific named test** | The decision text explicitly names a standardised cognitive instrument (e.g., MMSE, MoCA, or another named test). | *Example: “The file included an MMSE score of 18/30…”* |
| **Non-specific cognitive assessment** | The text refers to cognitive evaluation without naming a specific instrument. | *Examples: “neuropsychiatric assessment”; “psychometric evaluation”; “neuropsychological examination”; “cognitive test administered”; numerical score without test name.* |
| **No mention** | No visible reference to cognitive testing or assessment of any kind. |  |
|  | | |

| Coding rules | Only decision text scanned; full case file not accessible. Medical report text referenced in decision coded according to what the decision text reveals. Imaging findings (MRI, CT) and blood tests are NOT cognitive tests and are not coded here. |
| --- | --- |
| Critical note | Absence of a test name does NOT imply the test was not performed; Court of Cassation decisions are appellate summaries, not full case records. |
| Numerical score without test name | Coded as Non-specific cognitive assessment. |
| Study-wide statistics | Specific named test (MMSE): 3/1,031 (0.3%; 95% CI 0.1–0.8%); Non-specific: 101/1,031 (9.8%); No mention: 927/1,031 (89.9%). |
| Missing data | If full decision text not readable, noted in V15. |

| **V10 — Source of Medical Report** | |
| --- | --- |
| Variable type | Categorical (multiple values possible) |
| Definition | Institutional source of the medical report or dementia diagnosis referenced in the decision text. |
| Values | Forensic Medicine Institute (ATK) \| Clinical source (psychiatry / neurology / geriatrics / university) \| Not stated |
| Coding rules — ATK | ATK Ihtisas Kurulu or branch directorate reports coded as ATK. |
| Coding rules — Clinical source | Hospital psychiatry/neurology/geriatrics or university medical faculty report (non-ATK). |
| Coding rules — Multiple sources | Both coded; noted as “ATK + Hospital” or “Other / Mixed”. |
| Coding rules — Not stated | Report existence mentioned but institution not named; or report implicitly referenced. |
| Study-wide statistic | ATK: 484/1,031 (46.9%); Clinical: 158/1,031 (15.3%); Not stated: 389/1,031 (37.7%). |
| Missing data | Same as V07. |

| **V11 — Notarial Act** | |
| --- | --- |
| Variable type | Categorical (Yes / No / Not stated) |
| Definition | Whether the disputed legal act (sale, power of attorney, will, etc.) was executed before a notary. |
| Coding rules — Yes | Official (noter) will or deed; notarised power of attorney. |
| Coding rules — No | Handwritten will (does not require notarisation); informal written contract; title deed transfer (registered at land registry, not notary); court-executed act. |
| Coding rules — Not stated | Cannot be determined from decision text. |
| Notes | Notarially executed acts have implications for the notary’s duty to assess capacity — ethically significant dimension. Not applicable for criminal cases; left as N/A. |
| Missing data | N/A for criminal cases. |

| **V12 — Decision Outcome** | |
| --- | --- |
| Variable type | Categorical (4 levels) |
| Definition | Result of the Court of Cassation’s review. |
| Values | Reversal (Bozma) \| Approval (Onama) \| Partial \| Other |
| Coding rules | “Corrective approval” (düzelterek onama) → Approval. “Partial reversal” (kısmi bozma) → Partial. “Reversal for the benefit of law” (kanun yararına bozma) → Reversal (noted in V15). Reversal of a resistance ruling (direnme kararının bozulması) → Reversal. Remand / procedural referral (geri çevirme) → Other. |
| Study-wide statistics | Reversal: 524 (50.8%); Approval: 446 (43.3%); Partial: 29 (2.8%); Other: 32 (3.1%). |
| Missing data | None. |

| **V13 — Outcome Favourable to Patient** | |
| --- | --- |
| Variable type | Categorical (Favourable / Unfavourable / Indeterminate) |
| Definition | Whether the Court of Cassation’s decision outcome serves the interests of the person with dementia or their legal representatives. |
| Coding rules — Favourable | Decision protects or expands the rights of the person with dementia or their heirs/representatives. |
| Coding rules — Unfavourable | Decision upholds a finding adverse to the person with dementia or their heirs/representatives. |
| Coding rules — Indeterminate | Neutral, purely procedural decision; or outcome cannot be assessed without additional context. |
| Criminal cases | If defendant has dementia → defendant-favourable outcome coded as Favourable. If victim has dementia → victim-favourable outcome coded as Favourable. Role noted in V15. |
| Notes | This variable involves interpretive judgment and carries the highest coder-variability risk. Uncertain cases resolved by two-coder consensus and documented in V15. |
| Missing data | None. |

| **V14 — Summary of Legal Reasoning** | |
| --- | --- |
| Variable type | Text (free entry — qualitative content analysis) |
| Definition | Researcher-authored summary of the court’s legal reasoning, focusing on dementia-related content and the medico-legal interface. |
| Coding rules | Summary prepared by coder; based strictly on decision text. All dementia-related references and medico-legal reasoning captured. Personal data anonymised. |
| Notes | Quantitative coding category definitions (V06–V13) were developed iteratively from these summaries during the framework development phase. |
| Missing data | None. |

| **V15 — Notes** | |
| --- | --- |
| Variable type | Text (free entry — coder notes) |
| Definition | Additional coder annotations, inclusion/exclusion justifications, ambiguity resolutions, and audit trail entries. |
| Coding rules | Any variable-level ambiguity or special case documented here. Exclusion rationale noted for excluded decisions. Two-coder consensus outcomes documented. |
| Notes | Preserved for quality assurance and audit trail purposes. |
| Missing data | N/A |

**6. Derived Analytical Variables**

In addition to the 15 primary coded variables, the following binary and continuous variables were derived for analysis:

| **Derived variable** | **Values** | **Source / notes** |
| --- | --- | --- |
| **Subtype Specified (Binary)** | 1 = named subtype; 0 = general/unspecified | Derived from V07. Primary temporal outcome in logistic regression. |
| **Any Visible Cognitive Evidence (Binary; primary analytical outcome for temporal modelling)** | 1 = Specific OR Non-specific; 0 = No mention | Derived from V09. Used in parallel temporal regression (n = 104 events). |
| **Year (Continuous)** | 2005–2025 | Extracted from V01. Linear predictor in regression. |
| **Supreme Court Unit (Categorical)** | Civil / Criminal / General assembly | Derived from V01. Covariate in multivariable sensitivity model. |
| **Legal Context Category (Categorical)** | 7 categories | Derived from V06. Covariate in multivariable sensitivity model. |
|  | | |

**7. Illustrative Coding Examples**

The table below presents paraphrased decision text fragments with the corresponding V07 and V09 values to illustrate the application of coding rules.

| **Decision text (paraphrased)** | **V07 value** | **V09 value** |
| --- | --- | --- |
| Alzheimer’s disease diagnosis confirmed in ATK report; subtype explicitly named. | Alzheimer’s disease | No mention |
| Physician conducted cognitive evaluation; no test name stated. | General / unspecified | Non-specific cognitive assessment |
| MMSE score of 18/30 documented in case file. | General / unspecified | Specific named test (MMSE) |
| Decision uses only a generic dementia term (e.g., “bunama” — a Turkish colloquial/clinical equivalent) throughout. | General / unspecified | No mention |
| Neuropsychiatric assessment performed; result: capacity absent. | General / unspecified | Non-specific cognitive assessment |
| Vascular dementia explicitly stated in ATK report. | Vascular dementia | No mention |
| Parkinson and Alzheimer mentioned; clear subtype labelling present. | Alzheimer’s disease | No mention |
|  | | |

**8. Version Information**

| Version | Final revised version (Major Revision response) |
| --- | --- |
| Date | April 2026 |
| Corresponding author | Furkan İncebacak, MD |
| E-mail | furkanincebacak03@hotmail.com |
| Submitted as | Supplementary File 1 |
