# Supplementary material for: Dementia subtype specification and cognitive evidence visibility in Turkish Court of Cassation decisions: a document analysis of 1,031 decisions (2005–2025)
Source: BMC Med Ethics. 2026 May 21;27:161. doi: 10.1186/s12910-026-01480-w (PMC13464883; doi:10.1186/s12910-026-01480-w)
Supplement: Supplementary file 2 [file 12910_2026_1480_MOESM2_ESM.docx]

**Supplementary File 2**

**STROBE Statement — Checklist of Items That Should Be Included in Reports of Observational Studies**

Applied to: Cross-Sectional Document Analysis

Dementia Subtype Specification and Cognitive Evidence Visibility in Turkish Court of Cassation Decisions: A Document Analysis of 1,031 Decisions (2005–2025)

This STROBE checklist was completed in accordance with: von Elm E et al. The Strengthening the Reporting of Observational Studies in Epidemiology (STROBE) Statement: Guidelines for Reporting Observational Studies. BMJ. 2007;335:806–808. doi:10.1136/bmj.39335.541782.AD.

All items applicable to cross-sectional observational studies are addressed below. Items specific to cohort or case-control designs are noted as not applicable. The 'Location in MS' column refers to the submitted manuscript.

**STROBE Checklist**

| **Item No.** | **STROBE Recommendation** | **✓** | **Location in MS** | **Study-Specific Response** |
| --- | --- | --- | --- | --- |
| **TITLE AND ABSTRACT** | | | | |
| **1** | (a) Indicate the study's design with a commonly used term in the title or the abstract. | **✓** | Title; Abstract | Title states 'Document Analysis'; Abstract Methods state 'retrospective, cross-sectional, document-based analysis'. |
| **1** | (b) Provide in the abstract an informative and balanced summary of what was done and what was found. | **✓** | Abstract | Structured abstract (Background, Methods, Results, Conclusions) provided according to BMC Medical Ethics format. Primary outcomes, sample size, key results, and ethical implications reported. |
| **INTRODUCTION** | | | | |
| **2** | Background/rationale — Explain the scientific background and rationale for the investigation being reported. | **✓** | Introduction (§1) | Introduction reviews clinical relevance of dementia subtype differentiation, standardized cognitive testing, and their medico-legal significance. Gap in quantitative evidence on Turkish appellate courts is explicitly stated. |
| **3** | Objectives — State specific objectives, including any pre-specified hypotheses. | **✓** | Introduction (§1, final ¶) | Three research questions explicitly stated: (1) frequency of dementia subtype specification, (2) visibility of any cognitive evidence in decision texts, including either specific named tests or non-specific references to cognitive assessment, and (3) temporal patterns in subtype visibility and cognitive evidence visibility over 2005–2025. |
| **METHODS** | | | | |
| **4** | Study design — Present key elements of study design early in the paper. | **✓** | Methods (§2.1) | Retrospective, cross-sectional, document-based design within empirical legal studies framework; mixed-methods rationale stated (§2.1). |
| **5** | Setting — Describe the setting, locations, and relevant dates, including periods of recruitment, exposure, follow-up, and data collection. | **✓** | Methods (§2.2) | Data source: Yargıtay Karar Arama system (publicly accessible). Period: 2005–2025. URL provided. Document analysis start date and completion date noted in §2.2. |
| **6** | Participants — (a) Cohort/case-control: give eligibility criteria and sources and methods of selection. (b) Cross-sectional: give eligibility criteria, and the sources and methods of selection of participants. | **✓** | Methods (§2.2, §2.3) | Unit of analysis is Court of Cassation decisions (not human participants). Inclusion criteria: dementia/cognition directly relevant to legal determination. Exclusion criteria: keyword appears only incidentally (§2.3). Flowchart: 1,180 identified → 149 excluded → 1,031 included. |
| **7** | Variables — Clearly define all outcomes, exposures, predictors, potential confounders, and effect modifiers. Give diagnostic criteria, if applicable. | **✓** | Methods (§2.4); Supplementary File 1 | V07 (dementia subtype visibility) and V09 (cognitive evidence visibility) are defined as the two primary outcomes. V09 was coded in three levels: no mention, non-specific cognitive assessment, and specific named test. All 15 variables operationally defined in Supplementary File 1 (Codebook). |
| **8** | Data sources/measurement — For each variable of interest, give sources of data and details of methods of assessment (measurement). Describe comparability of assessment methods if there is more than one group. | **✓** | Methods (§2.2, §2.4); Supp. File 1 | All data derived from decision texts. Coding rules specified in Codebook (Supp. File 1). Keyword-based text identification followed by human coding. Both outcomes coded from same text, ensuring comparability. |
| **9** | Bias — Describe any efforts to address potential sources of bias. | **✓** | Methods (§2.2); Discussion (Limitations) | Search strategy bias (Alzheimer-specific keyword) acknowledged in §2.2 and Limitations. Retrieval lag for 2024–2025 noted. Coding ambiguity addressed through consensus procedure (Supp. File 1 §3). Limitation of single-source text coding acknowledged. |
| **10** | Study size — Explain how the study size was arrived at. | **✓** | Methods (§2.2, §2.3) | Sample determined by exhaustive keyword search of publicly accessible database during 2005–2025. No a priori power calculation applicable (descriptive/observational document analysis). Final n=1,031 after screening. |
| **11** | Quantitative variables — Explain how quantitative variables were handled in the analyses. If applicable, describe which groupings were chosen and why. | **✓** | Methods (§2.5 implicitly); Results (§3.2) | The three-level cognitive evidence visibility variable (no mention, non-specific cognitive assessment, specific named test) was dichotomised for temporal modelling as any visible cognitive evidence present vs. absent. Decision year treated as continuous linear predictor in logistic regression. Grouping rationale discussed in Results and limitations section. |
| **12** | Statistical methods — (a) Describe all statistical methods, including those used to control for confounding. (b) Describe any methods used to examine subgroups and interactions. (c) Explain how missing data were addressed. (d) Cross-sectional study: if applicable, describe analytical methods taking account of sampling strategy. (e) Describe any sensitivity analyses. | **✓** | Methods (§2.5); Results | (a) Descriptive statistics (frequencies, percentages, and 95% binomial confidence intervals for primary outcome proportions) were calculated. Univariable logistic regression was used to model temporal change in dementia subtype visibility, followed by a multivariable sensitivity model adjusting for Supreme Court unit and legal context. Model fit was evaluated using McFadden pseudo-R² and Hosmer–Lemeshow statistics. (b) Subgroup comparisons were examined using chi-square tests for dementia subtype visibility across Supreme Court units (Table 4) and for cognitive evidence visibility across legal contexts (Table 5), with cautious interpretation where sparse cells were present. (c) Missing data were minimal; cases with uncodeable year were excluded from year-based trend analysis only. (d) N/A (document analysis, not a probability sample). (e) A parallel temporal logistic regression was conducted for the broader binary outcome of any visible cognitive evidence (specific or non-specific combined; n = 104 events), which showed a statistically significant decrease over time (OR = 0.918 per year; 95% CI, 0.880–0.958; p < 0.001). |
| **RESULTS** | | | | |
| **13** | Participants — (a) Report numbers at each stage. (b) Consider use of a flow diagram. | **✓** | Methods (§2.3); Results (§3.1) | 1,180 identified; 149 excluded (keyword incidental or not legally determinative); 1,031 analyzed. Exclusion criteria applied and documented. Flow described in §2.3. |
| **14** | Descriptive data — (a) Give characteristics of study participants. (b) Indicate number of participants with missing data for each variable of interest. | **✓** | Results (§3.1); Table 1 | Table 1 provides full sample characteristics: Supreme Court unit, legal context, legal capacity assessment, medical report source, notarial act, decision outcome. Missing data noted where applicable. |
| **15** | Outcome data — Cross-sectional: Report numbers of outcome events or summary measures. | **✓** | Results (§3.2, §3.3); Tables 2–5 | Primary outcomes: subtype specified 478/1,031 (46.4%; 95% CI, 43.3–49.5%); any visible cognitive evidence 104/1,031 (10.1%; 95% CI, 8.3–12.1%); specific named test (MMSE) 3/1,031 (0.3%; 95% CI, 0.1–0.8%); non-specific cognitive assessment 101/1,031 (9.8%). 95% CIs provided for all primary proportions. |
| **16** | Main results — (a) Give unadjusted estimates and, if applicable, confounder-adjusted estimates. (b) Report category boundaries when continuous variables were categorized. (c) If relevant, consider translating estimates of relative risk into absolute risk for a meaningful time period. | **✓** | Results (§3.2); Table reporting | Main results included an unadjusted temporal odds ratio for dementia subtype visibility (OR = 1.062 per year; 95% CI, 1.034–1.091; p < 0.001), followed by an adjusted multivariable sensitivity model (adjusted OR = 1.055; 95% CI, 1.026–1.085; p < 0.001). A parallel logistic regression for any visible cognitive evidence showed a statistically significant decrease over time (OR = 0.918 per year; 95% CI, 0.880–0.958; p < 0.001). All OR estimates explicitly reframed as descriptive trend indicators in the limitations section. |
| **17** | Other analyses — Report other analyses done, e.g. analyses of subgroups and interactions, and sensitivity analyses. | **✓** | Results (§3.2–§3.4) | Additional analyses included chi-square comparisons of dementia subtype visibility by Supreme Court unit (Table 4; χ² = 6.53, p = 0.038) and cognitive evidence visibility by legal context (Table 5; χ² = 52.45, p < 0.001), a multivariable sensitivity model for subtype visibility, and a parallel temporal logistic regression for any visible cognitive evidence (OR = 0.918 per year; p < 0.001). |
| **DISCUSSION** | | | | |
| **18** | Key results — Summarise key results with reference to study objectives. | **✓** | Discussion (§4, opening ¶) | Opening paragraph of the Discussion summarises three main findings: (i) dementia subtype specification was visible in fewer than half of decisions, (ii) any visible cognitive evidence was uncommon and explicit naming of standardised instruments was exceptionally rare, and (iii) subtype visibility increased over time whereas any visible cognitive evidence decreased — two contrasting temporal trends. |
| **19** | Limitations — Discuss limitations of the study, taking into account sources of potential bias or imprecision. Discuss both direction and magnitude of any potential bias. | **✓** | Discussion (Limitations ¶) | Limitations paragraph addresses: (1) text-only data source (Cassation summaries ≠ full evidentiary record); (2) keyword retrieval bias; (3) publication lag; (4) absence of formal inter-rater reliability coefficients; (5) ATK reporting practices as alternative explanation; (6) limited international generalisability; (7) linearity assumption in logistic regression. Direction of each bias discussed. |
| **20** | Interpretation — Give a cautious overall interpretation of results considering objectives, limitations, multiplicity of analyses, and results from similar studies. | **✓** | Discussion (§4) | Causal language avoided throughout. Inferential overreach addressed: absence in decision text ≠ absence in full case file. Speculative interpretations explicitly flagged. Ethical implications positioned as interpretive hypotheses grounded in textual patterns. International literature cited (Näkki et al., 2024 [ref 21]). |
| **21** | Generalisability — Discuss the generalisability (external validity) of the study results. | **✓** | Discussion (Limitations ¶) | Turkish legal system's specific features (ATK dominance, procedural structure) noted as limiting direct international transfer. Acknowledged as explicit limitation. |
| **OTHER INFORMATION** | | | | |
| **22** | Funding — Give the source of funding and the role of the funders for the present study and, if applicable, for the original study on which the present article is based. | **✓** | Declarations | Funding section states: 'The authors received no specific funding for this work.' |

**Notes on Checklist Adaptation**

| **Note** | **Detail** |
| --- | --- |
| **Design** | This study uses a cross-sectional document analysis design; accordingly, STROBE items for cohort and case-control studies (e.g., matching, follow-up, loss to follow-up) are not applicable and have been omitted from this checklist. |
| **Participants** | The 'participants' in this study are judicial decisions (n=1,031), not human subjects. STROBE items referencing participants have been adapted accordingly: 'eligibility criteria' = inclusion/exclusion criteria for decisions. |
| **Ethics** | No human participants were involved. Data derived exclusively from publicly accessible, anonymised court decisions. Ethics committee approval was not required (confirmed in Declarations section). |
| **Software** | Statistical analysis performed in Python 3.x (pandas, scipy). Logistic regression: statsmodels library. Confidence intervals: Wilson score method for proportions <5%. |

**Compliance Summary**

| **Category** | **Items Applicable** | **Items Compliant** | **Compliance Rate** |
| --- | --- | --- | --- |
| Title and Abstract | 2 | 2 | **100%** |
| Introduction | 2 | 2 | **100%** |
| Methods | 9 | 9 | **100%** |
| Results | 5 | 5 | **100%** |
| Discussion | 4 | 4 | **100%** |
| Other Information | 1 | 1 | **100%** |
| **TOTAL** | **23** | **23** | **100%** |

Author declaration: All applicable STROBE items have been addressed in the submitted manuscript. The completed checklist is submitted as Supplementary File 2 per BMC Medical Ethics submission requirements.

**Corresponding Author: Furkan İncebacak, MD**

Email: furkanincebacak03@hotmail.com

Date: April 2026
