# Supplementary material for: Dementia subtype specification and cognitive evidence visibility in Turkish Court of Cassation decisions: a document analysis of 1,031 decisions (2005–2025)
Source: BMC Med Ethics. 2026 May 21;27:161. doi: 10.1186/s12910-026-01480-w (PMC13464883; doi:10.1186/s12910-026-01480-w)
Supplement: Supplementary file 3 [file 12910_2026_1480_MOESM3_ESM.docx]

**Supplementary Table S3**

*Year-by-year distribution of included decisions, dementia subtype visibility, and any visible cognitive evidence in Turkish Court of Cassation decisions (2005–2025).*

| **Year** | **Total n** | **Subtype specified n (%)** | **Any visible cognitive evidence n (%)** |
| --- | --- | --- | --- |
| 2005 | 1 | 0 (0.0%) | 0 (0.0%) |
| 2006 | 5 | 2 (40.0%) | 0 (0.0%) |
| 2007 | 12 | 4 (33.3%) | 0 (0.0%) |
| 2008 | 9 | 1 (11.1%) | 2 (22.2%) |
| 2009 | 11 | 3 (27.3%) | 3 (27.3%) |
| 2010 | 20 | 4 (20.0%) | 5 (25.0%) |
| 2011 | 34 | 9 (26.5%) | 6 (17.6%) |
| 2012 | 48 | 21 (43.8%) | 15 (31.2%) |
| 2013 | 66 | 21 (31.8%) | 6 (9.1%) |
| 2014 | 84 | 38 (45.2%) | 5 (6.0%) |
| 2015 | 94 | 43 (45.7%) | 11 (11.7%) |
| 2016 | 58 | 18 (31.0%) | 3 (5.2%) |
| 2017 | 61 | 32 (52.5%) | 7 (11.5%) |
| 2018 | 54 | 28 (51.9%) | 5 (9.3%) |
| 2019 | 47 | 27 (57.4%) | 5 (10.6%) |
| 2020 | 52 | 24 (46.2%) | 12 (23.1%) |
| 2021 | 54 | 31 (57.4%) | 7 (13.0%) |
| 2022 | 85 | 45 (52.9%) | 3 (3.5%) |
| 2023 | 86 | 43 (50.0%) | 1 (1.2%) |
| 2024 | 106 | 52 (49.1%) | 6 (5.7%) |
| 2025 | 44 | 32 (72.7%) | 2 (4.5%) |
| Total | 1031 | 478 (46.4%) | 104 (10.1%) |

**Note.** Year was derived from the decision-year field in the original coded Excel dataset (first four-digit year appearing in the decision header field). “Subtype specified” refers to decisions explicitly naming a clinically recognized dementia subtype, coded directly from the Excel field “Demans Türü” using the same manuscript classification rules: Alzheimer’s disease if “Alzheimer” appeared anywhere in the field; vascular dementia if the field began with an explicit vascular label; frontotemporal/frontal-type dementia if the field contained an explicit frontal/frontotemporal label; Parkinson’s disease dementia/parkinsonism plus dementia if “Parkinson” appeared without “Alzheimer”; otherwise general/unspecified. “Any visible cognitive evidence” was coded directly from the Excel field “Raporda Kullanılan Testler” as either a specific named test or a non-specific cognitive assessment reference, reproducing the manuscript totals of 478 subtype-specified decisions and 104 decisions with any visible cognitive evidence.
